# Climate adaptation through farmer choice: Understanding preferences for rice varieties in coastal Bangladesh

**DOI:** 10.1371/journal.pone.0347541

**Published:** 2026-04-29

**Authors:** Jaba Rani Sarker, John Rolfe, Jayanath Ananda

**Affiliations:** 1 School of Business and Law, Central Queensland University, Rockhampton, Queensland, Australia; 2 Department of Agricultural Economics, Gazipur Agricultural University, Gazipur, Bangladesh; 3 School of Business and Law, Central Queensland University, Melbourne Victoria, Australia; Gannon University, UNITED STATES OF AMERICA

## Abstract

The impact of environmental issues on agricultural livelihoods in Bangladesh's coastal regions—where traditional rice varieties are susceptible to salinity—is best illustrated by climate change-induced saltwater intrusion. Smallholder farmers have yet to adopt salt-tolerant rice varieties, despite the fact that they provide a technological intervention for climate adaptation. This underscores the disconnect between agricultural innovation and practical application. Farmers’ preferences for implementing these climate-resilient varieties are investigated in this study using a discrete choice experiment, which also looks at how contextual, institutional, and socio-economic factors influence technology adoption choices. Utilizing Random Parameter Logit and Latent Class Models, findings reveal three distinct farmer segments with varying willingness to pay for salt-tolerant varieties, influenced by age, savings, and land size. Post-hoc chi-square analysis reveals these preference segments are distributed evenly across districts (p = 0.822), indicating preference heterogeneity reflects individual-level differences rather than geographic salinity patterns. Each class from LCM represents the choice responses quite differently with different attributes. The majority of respondents belong to class 3 (46.5%), followed by class 1 (33.2%) and class 2 (20.3%). The largest group (46.5%) demonstrates strong preference for salt-tolerant varieties, while others prioritize different combinations of yield, profitability, and labor considerations. These results suggest that agricultural extension programs should implement differentiated strategies targeting each farmer segment, with subsidized seed distribution for resource-constrained farmers, demonstration plots for risk-averse adopters, and premium variety options for profit-oriented farmers. By highlighting the significance of context-specific approaches to promoting climate-resilient agricultural technologies and informing targeted policy interventions, these insights enable vulnerable coastal communities to achieve their broader development goals, including poverty alleviation, climate adaptation, and sustainable agricultural transformation.

## 1. Introduction

Climate change represents one of the most significant challenges to development in the 21st century, disproportionately affecting vulnerable populations in developing countries and threatening progress toward poverty alleviation and sustainable development goals [1,2]. Bangladesh demonstrates this crisis, where climate-induced environmental changes intersect with socio-economic vulnerabilities to create complex development challenges that demand context-sensitive interventions [3,4].

The southern coastal regions of Bangladesh present a case study of how global climate processes interact with local development contexts to shape agricultural livelihoods and food security outcomes [5]. Here, climate change exacerbates existing socio-economic vulnerabilities, threatening the development progress of millions of rural households [6]. About 70% of Bangladesh's rural population depends on agriculture for their livelihoods [7], so increasing agricultural productivity in these climate-vulnerable areas is essential for reducing poverty and promoting sustainable development [8].

Rice cultivation in Bangladesh is more than agricultural production—it represents a fundamental pillar of human well-being, food security, and economic development. As the staple food for roughly 15 million farming families, rice accounts for nearly half of the country's agricultural GDP and provides two-thirds of per capita daily calorie intake, making it indispensable for public health and nutrition security [9]. Rice is the predominant food crop [10], cultivated across three main seasons, with Boro rice contributing approximately 54% of total production [11]. However, this production faces threats from climate-induced salinity intrusion, particularly affecting Boro cultivation and perpetuating rural poverty in saline-prone areas [12,13].

The development and dissemination of climate-smart agricultural technologies represents a promising pathway for addressing the intersection of climate change and rural development challenges [14]. Salt-tolerant rice varieties, in particular, have emerged as a vital technological intervention for building resilience against salinity intrusion—a challenge that demonstrates how global environmental processes manifest as local development constraints [15]. These multi-scale development efforts have yielded significant technological advances [16]. Despite these advances, however, a persistent disconnect remains between agricultural research and its practical application. This is evident in the limited adoption of salt-tolerant varieties by coastal farmers, where traditional rice varieties still dominate agricultural landscapes [17]. The resulting adoption gap highlights a broader development challenge: ensuring that technological interventions effectively reach and benefit the vulnerable populations for whom they are intended [18,19].

To adapt to these climate-induced stresses and ensure sustainable food production, the adoption of climate-smart agricultural practices and resilient rice varieties has become a priority [20]. Notably, developing salt-tolerant rice varieties is recognized as a vital step in building resilience against salinity intrusion, a challenge exacerbated by climate change and inefficient land management practices [21,22]. Several international and national research organizations, including the Bangladesh Rice Research Institute (BRRI) and the Bangladesh Institute of Nuclear Agriculture (BINA), have made significant advances in this area. These efforts have produced varieties capable of withstanding salinity levels of 10–12 dS m ⁻ ¹ at the seedling stage, offering promising options for farming in coastal environments [23,24]. Nevertheless, scaling adoption at the farm level will require strengthened extension services, improved seed access, farmer awareness initiatives, and targeted policy support to bridge the gap between research innovations and practical uptake [25,26].

Several studies have emphasized the importance of understanding farmers’ preferences for rice traits to facilitate widespread adoption of new varieties. Recent applications of stated preferences methods such as discrete choice experiments and best-worst scaling have been instrumental in uncovering the heterogeneity of farmers’ preferences, revealing that attributes like yield potential, seed cost, and labor requirements significantly influence decision-making [27,28]. For instance, in India, preferences for drought- and flood-tolerant varieties reflect a focus on yield stability and resilience [27]. At the same time, research in Kenya shows that farmers prioritise early maturity and seed weight when selecting rice varieties [29]. Farmers’ preferences for rice varieties are primarily driven by traits such as potential yield, maturity, pest and disease resistance, and seed longevity, with distinct groups differing in their emphasis on factors like price sensitivity, environmental concerns, and access to resources based on their socio-economic and contextual characteristics [30]. Socio-economic factors such as landholding size, household income, education level, and access to extension services also play critical roles. For example, farmers with larger farm sizes have a greater tendency to adopt improved rice varieties [31]. However, these studies often overlook salinity risk, a particularly pressing concern for coastal farmers in Bangladesh.

Salinity adversely impacts crop growth by hindering seed germination, disrupting nutrient uptake, and inducing physiological stress, ultimately threatening both food security and farmers’ livelihoods [32–34]. To address this, the development and dissemination of salt-tolerant rice are crucial strategies promoted by both government and international agencies, aiming to reduce salinity-related yield losses and promote sustainable agriculture [35,36]. Nevertheless, adoption rates for salt-tolerant varieties remain low, as farmers tend to prefer familiar traditional types that can somewhat withstand floods and salinity [37,38].

This persistent adoption gap highlights a critical need for a more sophisticated understanding of how contextual factors interact with technological interventions to shape development outcomes in climate-vulnerable communities [39]. Recognizing farmer heterogeneity emerges as essential for designing effective policies and interventions that can successfully promote climate adaptation while addressing poverty alleviation and sustainable development objectives [40].

Despite the recognized importance of salt-tolerant rice varieties for coastal Bangladesh, previous research in the country has primarily focused on agronomic performance and breeding programs rather than farmer adoption behavior and preferences. Studies by Rahman et al. [41] and Dasgupta et al. [42] have documented the technical effectiveness of salt-tolerant varieties and their economic impacts under saline conditions. However, these studies largely overlooked the socio-economic and behavioral factors that influence farmers’ adoption decisions. Similarly, while Rahman et al. [43] identified novel quantitative trait loci (QTL) for salinity tolerance using Bangladeshi landraces, their analysis was limited to genetic mapping without exploring farmer preferences for specific varietal attributes.

More recently, Islam et al. [44] examined farmers’ perceptions of salinity tolerance in southwestern Bangladesh, but their focus was primarily on technical performance rather than willingness-to-pay for adaptive technologies. Debsharma et al. [45] developed climate-resilient varieties (BRRI dhan97 and BRRI dhan99) but did not investigate farmer preferences for these innovations. This represents a significant research gap, as understanding farmer preferences and their heterogeneity is crucial for designing effective dissemination strategies.

Furthermore, while international literature has extensively employed discrete choice experiments to understand farmer preferences for crop varieties [27–30], such rigorous stated preference methods have been underutilized in the Bangladeshi context, particularly for salt-tolerant rice varieties. The present study addresses these research gaps by being the first to employ discrete choice experiments specifically to understand farmer preferences for salt-tolerant rice varieties in coastal Bangladesh.

The research makes several important contributions to the development literature. First, it provides rigorous empirical evidence on farmer preferences in a climate-vulnerable region facing acute development challenges [46,47]. Second, it demonstrates how advanced econometric methods can inform context-sensitive policy design for agricultural development and climate adaptation [48]. Third, it offers practical insights for scaling climate-resilient technologies in ways that address poverty alleviation and food security objectives [49,50]. Finally, it contributes to a broader understanding of how global environmental changes interact with local development contexts to shape technology adoption patterns and development outcomes in developing countries [51].

The remainder of the article is organised as follows. Section 2 presents methods and explains the choice experiments in detail, and the empirical model. Section 3 presents the results of the analysis, the discussion of findings is presented in Section 4, and Section 5 gives a summary of the main findings and policy recommendations.

## 2. Methods

The study used a discrete choice experiment to examine farmer preferences for adopting newer rice varieties and to explore heterogeneity in these preferences. Choice experiments have been widely used in the agricultural and environmental economics literature, and their use in development economics is gaining popularity. Over the past decades, Discrete Choice Experiments (DCEs) have been frequently used to explain decision-making in high-income countries in agriculture [52–56], industry [57], environment [58–60] and health [61]. However, there are comparatively few examples of DCEs being utilized in developing countries [62].

In developing countries, DCEs have been used to measure farmers’ preferences in drought risk management [27,63,64], drought and flood risk management [28,65], climate change adaptation [66,67], conservation agriculture [68], alternative crop intensification [69], farmland consolidation [70]; intercropping system [71]; and agricultural technology adoption [72]. Specifically, these studies addressed how to manage and mitigate drought risk in Bangladesh, drought risk in Vietnam, drought and flood risk in India, and how to apply conservation agriculture strategies and the heterogeneity in preferences of the intercropping system in Malawi. Farmers’ WTP for improved climate service has been investigated by Tesfaye et al. [73] using discrete choice experiments. The application of DCEs has been noted in the management of environmental resources [73]. In addition, Othman et al. [74] demonstrate that choice modelling is an effective approach for capturing stakeholder preferences in environmental and resource management decisions, while Furuya et al. [75] identified strong farmer demand for weather index insurance in Myanmar - particularly for risks such as cyclone landfall, flooding, and drought. Bannor et al. [76] also identified that poultry farmers are willing to pay more to secure their farms.

The article reports the application of a discrete choice experiment to farmers in Bangladesh to evaluate their preferences for adopting salt-tolerant varieties of rice. The hypotheses of interest for the present study are:

a) Farmers have diverse preferences on Traditional rice varieties (TRVs) and High Yielding Varieties (HYVs) or Salt Tolerant Varieties (STVs) of rice.
b) Farmers’ preferences for HYVs of rice are different from those for salt-tolerant varieties in coastal Bangladesh.
c) Farmers of different classes (in the latent class model) have diverse preferences for newer rice varieties.
d) Socio-economic factors influence the adoption of different newer rice varieties in a different manner.

**Fig 1:**
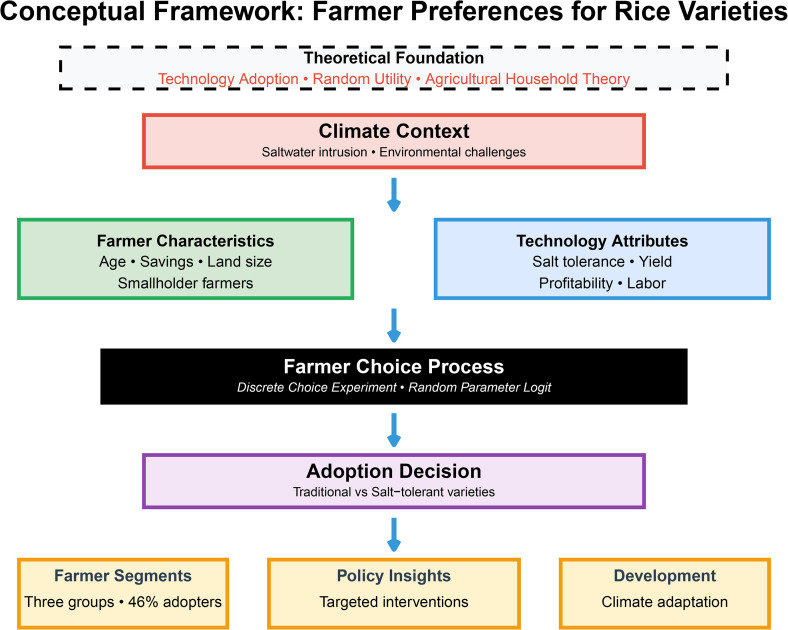
The conceptual framework of the study.

The conceptual framework of the study is shown in Fig 1.

### 2.1. Discrete choice experiments

Discrete choice experiments (DCE), also termed as choice modelling, are a widely used non-market valuation technique used to assess stated preferences when actual market transactions are not observable [50]. DCEs involve participants making choices between similar profiles [77], allowing researchers to examine responses to hypothetical scenarios based on selected attributes. DCEs value economic benefits or costs that are not revealed in markets [71] and assess demands for market goods that are not yet available or where market data is not available. DCEs avoid common problems in revealed preference analysis, such as attribute-level invariance and multicollinearity [71], while being more cost-effective than market data analysis [78].

The study identifies rice farmers’ livelihood choices in Bangladesh under increasing salinity, an under-researched topic where revealed data is not yet available. The problem involves various factors that farmers could consider, including continuing to use current rice varieties or adopting new varieties. The DCE framework offers respondents different alternatives and asks them to choose the option that provides the highest level of utility for them [54]. DCE is an appropriate method for capturing farmers’ preferences regarding the adoption of newer rice varieties, as it gathers individual data on scenarios that can be applied to future contexts, especially when they are dealing with challenges in rice production due to salinity intrusion.

The experiments determine farmers’ decision-making processes regarding newer rice varieties and resulting model parameters. In DCEs, respondents choose preferred options from attribute alternatives across multiple tasks [79], with attribute levels varied to create scenario diversity. Careful consideration of the attributes, their levels, and the number of choice tasks presented to respondents is part of the process of designing a DCE. The involvement of payment attributes is normally presented to the respondents in monetary terms to make it easily understandable. Attributes and levels need to be presented in interpretable, accurate, and measurable terms [80].

#### 2.1.1. Designing the Discrete Choice Experiments (DCEs).

The choice experiment was framed to farmers as a choice between newer rice varieties and different levels of input and output consequences. The first step was framing the overall dimensions to be a choice between the status quo option (continue to grow current varieties of rice) or move to newer varieties. Saline-tolerant variety was presented as a categorical seed type option in our choice experiment, reflecting how farmers evaluate varietal choices in practice. This design reflects the real-world information environment in Bangladesh where extension services and seed systems promote varieties categorically as “salt-tolerant” or “not salt-tolerant” without specifying precise tolerance thresholds. Farmers make binary adoption decisions rather than selecting varieties with specific tolerance levels matched to their salinity conditions. This approach allows us to examine whether coastal farmers value salt tolerance as a varietal attribute and how they trade off this characteristic against other factors such as yield, cost, and labor requirements. We held multiple focus groups discussion with Boro rice farmers in Bangladesh's coastal regions, consulted with Upazilla Agricultural Officers, and reviewed existing literature [27,28] to identify key attributes: yield of rice, seed price, labor supply, and rice output price. The pilot survey involved discussions with extension service personnel to refine DCE questions, followed by focus group discussions with farmers from four districts. During these discussions, different rice varieties (salt-tolerant, high-yielding, pest-resistant, and current varieties) were presented, with pest-resistant varieties ultimately excluded due to lower farmer importance. Making sure the respondents could understand and identify with the options was our goal. To achieve this, we translated the survey choice sets into Bengali (the native language), and the data collectors used the same language when interviewing the participants. Additionally, we used visual aids (when necessary) to enhance the comprehension of the alternatives. Attribute levels were designed for realism and sufficient variation. For example, seed price levels were set at 5%, 10%, 15%, and 20% above current varieties, validated during pilot testing for appropriateness.

Producer preferences decrease with increasing output price risk [81]. Thus, the output price is related to farmers’ production choices. If farmers do not receive a better price, they may be reluctant to continue cultivating certain crops, directly impacting profitability and cultivation choices. This attribute allowed assessment of producers’ price sensitivity and willingness to accept price fluctuations.

**Fig 2:**
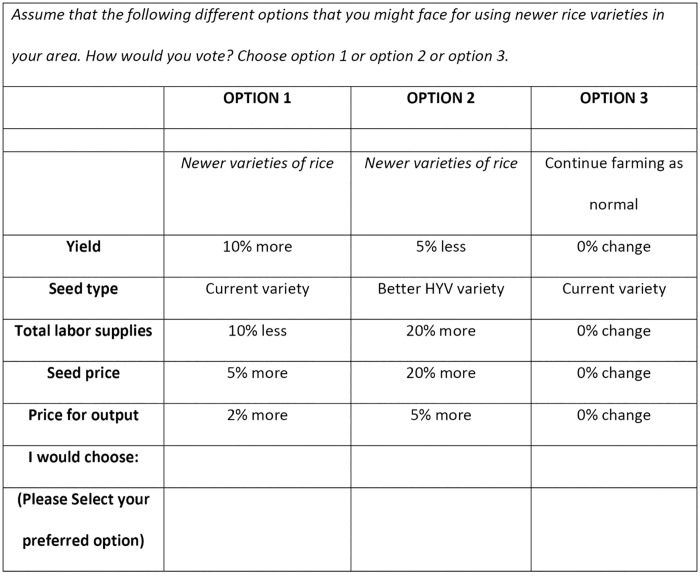
A sample choice set from the CM experiment.

The different attribute levels that appeared in the choice sets are outlined in Table 1, and an example choice set is presented in Fig 2. To set the attribute levels, two basic considerations were applied. First, the levels needed to be as realistic as possible, based on current conditions. Second, to elicit variations in response from farmers, distinct differences between the different levels of each attribute were desirable. Before completing the choice sets, respondents were reminded that there were likely to be a range of different factors that they might consider in their decision to accept an option.

**Table 1:** Attributes and levels used in the experiment.

	Attribute	Variable type	Level	Base
**1**	**Yield**	Continuous	1 = 5% less	
			2 = 0% change	0% change
			3 = 5% more	
			4 = 10% more	
2	**Seed type**	Categorical	1 = Current variety	Current variety
			2 = Better HYV variety	
			3 = Better Saline-tolerant variety	
3	**Total labor supplied**	Continuous	1 = 10% less	0% change
			2 = 0% change	
			3 = 10% more	
			4 = 20% more	
4	**Price of Seed input**	Continuous	1 = 5% more	0% change
			2 = 10% more	
			3 = 15% more	
			4 = 20% more	
**5**	**Price of output**	Continuous	1 = 0% change	0% change
			2 = 2% more	
			3 = 5% more	
			4 = 10% more	

Our discrete choice experiment was designed with five attributes, each with four levels, which would generate 4^5 = 1,024 possible combinations in a full factorial design that would be cognitively overwhelming and practically infeasible for respondents. Following Hensher [82] and Meyerhoff et al. [83], who demonstrated that design dimensionality significantly affects choice consistency and behavioral responses in environmental contexts, we employed a Bayesian D-efficient experimental design using Ngene software [84,85], which differs fundamentally from the orthogonal design approach used by Tanaka et al. [86]. While Tanaka et al. [86] mechanically reduced 72 combinations to 24 choice sets using orthogonal design principles, our Bayesian D-efficient approach optimally selected 24 choice sets from the full 1,024 possible combinations based on statistical efficiency criteria rather than simple reduction rules.

The determination of 24 choice sets was based on several methodological considerations: (1) Statistical requirements – ensuring sufficient degrees of freedom to estimate all parameters with adequate precision, which for our five attributes with main effects required a minimum of 20 choice observations; (2) Design efficiency optimization: the Bayesian algorithm iteratively selects choice sets that maximize the determinant of the information matrix, with our algorithm determining the optimal number to be 24; (3) Cognitive feasibility: research shows respondents can effectively process 20–32 choice tasks when properly blocked [84]; and (4) Prior parameter uncertainty: Bayesian designs require fewer choice sets than orthogonal designs when reasonable priors are available. Unlike orthogonal designs that focus on attribute-level balance, D-efficient designs prioritize statistical precision by specifically selecting choice sets to minimize parameter estimate variance rather than simply maintaining mathematical orthogonality. This approach typically requires 20–40% fewer choice sets than orthogonal designs to achieve the same statistical power [87], explaining why our theoretically larger attribute space (1,024 vs. 72 combinations) resulted in the same number of final choice sets as Tanaka et al.’s study. To minimize respondent fatigue, we blocked the 24 choice sets into six groups of 4 choice sets each, with farmers randomly assigned to one block, ensuring each respondent completed a manageable number of tasks while the full experimental design captured all necessary variation across the sample.

### 2.2. Data analysis methods

The discrete choice experiments (DCEs) data were analysed using LIMDEP software. Three different statistical models were employed to identify meaningful results. Initially, we employed the traditional conditional logit model (MNL) to analyse farmers’ preferences for newer rice varieties. Although widely used, the conditional logit discrete-choice model has some limitations, particularly its independence of irrelevant alternative (IIA) property, which can result in counterintuitive outcomes. Thus, in the second stage of analysis, we applied the more flexible random parameter logit model (RPL). In the third stage, the study employed latent class models to identify into whether there were very different groups of farmers responding.

### 2.3. Empirical model

In line with conventional approaches to Discrete Choice Experiments (DCE), we operate within the framework of random utility theory, which considers decisions as occurring within a utility maximization context [88]. Suppose an individual (i) faces a set of (j) alternatives, denoted by (S), during a particular choice occasion (t). We define an unobservable, latent variable V^*^_ijt_ that represents the true utility associated with individual (i) selecting option (j) within choice set (S) at time (t). According to utility maximization principles, individual (i) will prefer alternative (j) if V*ijt exceeds the utility of all other options V*_iqt_ ∀q ≠ j. The actual utility perceived by the researcher, denoted as Vijt, differs from the latent utility but is known to be maximized when the chosen alternative corresponds to the greatest V*_ijt_ that is, $V_{ijt}=\text{1}\;if\;\overset{*}{\underset{ijt}{V}}=\text{max}(\overset{*}{\underset{i\text{1}t}{V}};\;\overset{*}{\underset{i\text{2}t}{V}};\ldots \ldots \ldots \ldots ;\overset{*}{\underset{iJt}{V}})$ and 0 otherwise.

Assuming that the utility derived from each alternative depends linearly on its attributes, the individual (i)’s latent utility function can be expressed as:


$$\overset{*}{\underset{ijt}{V}}=\;\overset{\prime }{\underset{ijt}{X}}\beta +\;\varepsilon_{ijt}$$ (1)

where X'_ijt_ is a vector representing the attributes of the (j)-th option for individual (i) in choice situation (t), (\β) is a vector of parameters indicating individual preferences (or attribute weights), and ε_ijt_ is a stochastic component capturing unobserved factors and measurement errors. We assume ε_ijt_ follows a Gumbel (Extreme Value Type I) distribution, and that these error terms are independent and identically distributed across individuals and choices. Under this assumption, the probability that individual (i) selects alternative (j) from set (S) at time (t)—based on the observed attribute levels—is modeled using the conditional logit model, which is estimated via maximum likelihood. This approach presumes homogeneous preferences across respondents and maintains the independence of irrelevant alternatives (IIA) assumption [89].

To account for variation in preferences across individuals, the random parameters logit (RPL) model is often employed. This model introduces random variations in the attribute coefficients, relaxing traditional IIA limitations and allowing for heterogeneity in tastes within the sample [48]. Following specifications outlined by Train [90], the probability that individual (i) chooses alternative (j) from the choice set (S) during occasion (t) is given by:


$$Prob\left(V_{ijt}\;=\;\text{1}|\overset{\prime }{\underset{i\text{1}t,\;}{X}}\overset{\prime }{\underset{i\text{2}t}{X}},\ldots \ldots .,\overset{\prime }{\underset{ilt}{X}},\Omega \right)=\;\int \frac{\text{exp}\left(\overset{\prime }{\underset{ijt}{X}}\beta_{i}\right)}{\sum_{q=\text{1}}^{Q}\text{exp}\left(\overset{\prime }{\underset{iqt}{X}}\beta_{i}\right)}f\left(\beta |\Omega \right)d\beta$$ (2)

where β_i_ represents a vector of parameters that vary randomly across individuals, and

/Ω/  characterizes the distribution governing these random parameters. The researcher can specify different distributional forms for these random coefficients and impose restrictions when relevant. In our case, the coefficients associated with the rice yield and rice seed price attributes were modeled as random parameters, while the coefficients for rice output price and total labor input were treated as fixed effects.

In order to classify farmers into groups with similar fundamental features, a latent class specification was also employed. In latent class analysis, f (β) is discrete, taking C distinct values [90]. The probability that farmer n selects option j in a given choice set S, unconditional on the class, is represented by


$$P_{njs}\;=\;\sum_{c=\text{1}}^{C}\frac{exp(\overset{\prime }{\underset{njs}{X}}\beta_{c}}{\sum_{k}exp(\overset{\prime }{\underset{nks}{X}}\beta_{c})}R_{nc}$$ (3)

where β_c_ is the specific parameter vector for class c, and R_nc_ is the probability that producer n falls into class c. This probability can be conditioned by a vector of household characteristics z_n_, and a coefficient vector corresponding to membership in class c, θ_c_, in a similar logit function:


$$R_{nc}\;=\;\frac{exp(\overset{\prime }{\underset{n}{z}}\theta_{c}}{\sum_{c}exp(\overset{\prime }{\underset{n}{z}}\theta_{c}}$$ (4)

We can therefore rewrite Equation (3) as


$$P_{njs}\;=\;\sum_{c=\text{1}}^{C}[\left(\frac{exp(\overset{\prime }{\underset{njs}{X}}\beta_{c}}{\sum_{k}exp(\overset{\prime }{\underset{nks}{X}}\beta_{c})}\right)\;\left(\frac{exp(\overset{\prime }{\underset{n}{z}}\theta_{c}}{\sum_{c}exp(\overset{\prime }{\underset{n}{z}}\theta_{c}}\right)]$$ (5)

The latent class analysis allows for the identification of groups of farmers that are heterogeneous across classes and homogenous within a group. This facilitates the identification of producers with similar preference structures, enabling policy recommendation that targets individual farmer groups and allows for the estimation of the tradeoffs that farmers make when choosing to adopt a given rice variety.

### 2.4. Variable construction

A binary age variable was created using 55 years as the cutoff, representing farmers in the later stages of their farming careers. This threshold aligns with observed patterns in agricultural adoption literature where older farmers (55+) exhibit lower technology adoption rates due to reduced physical capacity for intensive farm management, shorter planning horizons that diminish returns on long-term investments, and greater risk aversion toward unfamiliar technologies [91]. In our sample, 38% of farmers were aged 55 or above, providing sufficient variation for statistical analysis while capturing a meaningful life-stage transition relevant to adoption behavior.

Monthly household savings were coded as a binary variable (No savings and below Tk 5,000 = 1, 0 = Otherwise). This threshold identified households with limited financial capacity (19.5% of sample) distinct from the majority (80.5%) with moderate to higher liquidity. The Tk 5,000 cutoff represents approximately 8–12 days of agricultural labor income based on prevailing wage rates in coastal Bangladesh (Tk 400–600 per day) [92] and aligns with the minimum capital requirements for basic climate-adaptive investments.

### 2.5. Study area and data source

The study was conducted in the southwestern and southcentral coastal zone of Bangladesh, where salinity intrusion is an emerging issue. We selected study areas where rice production is currently active, as rice cultivation becomes economically unviable in severely saline areas (above 8–10 dS/m), leading farmers to abandon rice production entirely. Within this constraint, we chose farming districts with varying but manageable salinity levels (2–8 dS/m) to capture heterogeneity in farmers’ experiences with salinity stress while ensuring all respondents could meaningfully evaluate preferences for salt-tolerant varieties. This approach ensures our findings are applicable to areas where salt-tolerant rice varieties have the greatest potential impact—where rice production is threatened but not yet abandoned due to salinity intrusion. A multi-stage sampling technique was used where farming districts were selected that varied in the level of salinity and the availability of farming opportunities: three districts from the Khulna Division (Satkhira, Khulna, and Bagerhat) and one district from the Barisal Division (Patuakhali). Khulna Division encompasses multiple high-salinity districts where rice cultivation persists despite severe climate challenges. According to Akter et al. [93], Satkhira, Khulna, and Bagerhat are among the districts expected to face the most severe consequences from escalating river salinity due to climate change, necessitating inclusion of all three districts to capture intraregional diversity in salinity exposure and adaptive responses. Patuakhali, from the Barisal Division, was selected to represent the relatively lower-salinity yet climate-stressed rice systems of the eastern coast, providing important contrast to the high-salinity western districts. These four districts (Khulna, Satkhira, Bagerhat, and Patuakhali) are from the southern part of Bangladesh. One upazila was purposively selected from each district. At the next stage, two unions from each upazila were purposively selected in consultation with the local agricultural office. Thus, the field study was conducted in eight unions belonging to four upazilas of four districts.

While our multi-stage sampling deliberately selected districts with varying salinity levels (2–8 dS/m) to capture heterogeneity in farmers’ salinity exposure experiences, we did not incorporate district-level salinity variation as a moderating variable in the choice models. This methodological decision reflects our research focus: examining whether coastal farmers in salinity-affected regions value salt tolerance as a varietal attribute, rather than modeling how preference intensity varies continuously across the salinity gradient. This sampling strategy ensures our findings are representative of coastal farmers experiencing different degrees of salinity stress.

The four selected districts represent a gradient of salinity stress and adaptive capacity. Satkhira and Khulna face the most severe salinity intrusion (6–12 dS/m during dry season) and tidal flooding, limiting freshwater access and crop diversity [94]. Reduced freshwater flow from the Ganges due to upstream withdrawals exacerbates salinity in these western districts [95]. Bagerhat experiences moderate salinity levels (4–8 dS/m) with mixed rice–fish systems emerging as adaptation strategies, where approximately 86% of farmers practice integrated prawn-fish-rice farming in gher systems [96]. Patuakhali faces seasonal waterlogging during monsoon periods when high river water levels prevent adequate drainage yet achieves higher cropping intensity (up to 19.3 t/ha/year in intensified aus-aman-rabi systems) due to better access to freshwater irrigation from tidal channels and lower dry-season salinity (2–4 dS/m) compared to western districts [97]. These spatial variations represent the environmental context within which sampled farmers operate. While we describe this geographic heterogeneity for contextual understanding, our analysis identifies preference segments among coastal farmers generally rather than testing how district-level salinity moderates preferences. Post-hoc chi-square analysis reveals that the three farmer classes identified through our Latent Class Model are distributed relatively evenly across all districts (Table 5), indicating preference heterogeneity operates independently of geographic salinity patterns.

**Table 5:** Distribution of Latent Classes Across Districts and Salinity Zones.

District	Salinity Level	Class 1 n (%)	Class 2 n (%)	Class 3 n (%)	Total
Bagerhat	Moderate (4–8 dS/m)	16 (32%)	8 (16%)	26 (52%)	50 (100%)
Khulna	High (6–12 dS/m)	18 (36%)	13 (26%)	19 (38%)	50 (100%)
Patuakhali	Low (2–4 dS/m)	15 (30%)	11 (22%)	24 (48%)	50 (100%)
Satkhira	High (6–12 dS/m)	17 (34%)	9 (18%)	24 (48%)	50 (100%)
**Total**		**66 (33%)**	**41 (20.5%)**	**93 (46.5%)**	**200 (100%)**

*Note: Class 1 represents farmers with no significant preference for salt-tolerant varieties; Class 2 represents limited engagement farmers; Class 3 represents farmers with strong preferences for salt-tolerant varieties. Chi-square test of independence: χ² = 2.893, df = 6, p = 0.822.*

The area selection ensured our sample represents coastal farmers for whom salt-tolerant varieties are relevant. While districts varied in salinity (2–8 dS/m), our analytical focus was identifying preference segments within this coastal population rather than testing district-level effects. Two villages were selected from one union. For each village, a list of rice farmers was collected from the local agricultural office, and respondents were selected randomly from this list. In the final stage, 50 households were selected from each union (25 respondents from each village), and thus the study has interviewed 200 households. The selected farmers were boro rice growers. Data from the 200 farmers was collected by face-to-face interviews with a survey questionnaire between the 15 June 2022–14 August 2022. For the choice experiment, this yielded 800 observations (200 households*4 choice sets). Verbal consent to conduct the interviews for the purposes of this research was obtained from all respondents during data collection, who were fully informed about the purposes of this research and how their responses would be used.

### 2.6. Ethical approval

The research was carried out in accordance with conditions of approval from the Central Queensland University, Australia Human Research Ethics Committee (ethics approval number 0000023083).

A written consent form, approved by the IRB (the Human Research Ethics Committee, CQ University- HREC reference number: 23083), was presented to the respondents. Then, verbal informed consent was obtained from all rice farmer participants prior to interviews, conducted in Bengali to ensure full comprehension. The consent process included explanation of the study purpose, voluntary participation, confidentiality measures, and participants’ right to withdraw at any time. Participants confirmed their understanding and agreement verbally before proceeding with data collection. Verbal consent was deemed appropriate given the rural context, literacy considerations, and cultural norms of the farming communities studied.

## 3. Results

The results of a conditional logit (MNL) model are presented in Table 2. All variables are treated as continuous except for Seed Type, which has been modelled as a dummy variable for each level, with Seed Type 1 omitted to act as a base. All attributes in the model except rice seed price and seed type 3 (better salt-tolerant variety) are statistically significant at conventional levels, and their signs are as expected *a priori*. Higher rice yields and higher output prices have positive coefficients, whereas higher seed prices and higher labor inputs have negative signs.

**Table 2:** Results from the conditional logit (MNL model).

Variables	Coefficient
Yield of rice	0.282^a^ (0.020)
Seed price of rice	−0.040 (0.029)
Seed type 2(Better HYV rice)	−3.166^a^ (0.685)
Seed type 3(Better salt-tolerant rice)	−0.748 (0.573)
Total labor supplied	−0.040^a^ (0.014)
Rice output price	0.116^a^ (0.022)
Seed type 2 * Rice seed price	0.197^a^ (0.048)
Seed type 3 * Rice seed price	0.074^c^ (0.040)
Total labor supplied * Seed type 2	0.047^c^ (0.025)
Total labor supplied * Seed type 3	0.041^c^ (0.021)
ASC(Constant)	1.986^a^ (0.518)
Age _dummy (>55 years = 1, 0 = Otherwise)	−0.546^b^ (0.245)
Savings_dummy (No savings and below tk 5k = 1, 0 = Otherwise)	−0.901^a^ (0.230)
Total rice land (in decimal)	−0.005^a^ (0.001)
**Model statistics**	
Number of observations	800
Log likelihood function	−494.446
AIC	1014.9
AIC/N	1.271
McFadden’s R-sqrd	0.416

*Note: Standard errors are reported in parentheses.*

^a^ *Significance at 1%*

^b^ *Significance at 5%*

^c^ *Significance at 10%*

The constant term and the enterprise and socio-demographic factors have been coded to represent the status quo option. The positive coefficient for the constant term thus indicates an underlying preference to continue growing traditional varieties of rice. Socio-demographic factors such as age, savings, and total rice land are also negative and highly significant, indicating that younger farmers, those with lower savings, and those with smaller land parcels are more likely to choose the status quo option. The overall fit of the model, as measured by McFadden’s r^2^, is also acceptable by conventional standards used to describe probabilistic discrete choice models [98].

Table 3 reports a random parameter logit model with a similar structure, but with the coefficients for yield of rice and price of rice seed assumed to follow a normal distribution across respondents. Tests revealed that the strongest model fits were achieved when only these two attributes were randomized. The model includes coefficients from the main effects and some key interactions, which allow richer analytical insights into farmers’ preferences.

**Table 3:** Random Parameters (Mixed) Multinomial Logit Model.

Variables	Coefficient
**Random parameters in utility functions**	
Yield of rice	0.504^a^ (0.052)
Price of rice seed	−0.100^b^ (0.044)
**Nonrandom parameters in utility functions**	
Seed type 2 (Better HYV rice)	−4.911^a^ (1.009)
Seed type 3 (Better salt-tolerant rice)	−1.999^b^ (0.835)
Total labor supplied	−0.059^a^ (0.021)
Rice output price	0.188^a^ (0.034)
Seed type 2 * Rice seed price	0.280^a^ (0.067)
Seed type 3 * Rice seed price	0.156^b^ (0.059)
Total labor supplied * Seed type 2	0.065^c^ (0.035)
Total labor supplied * Seed type 3	0.078^b^ (0.030)
ASC(Constant)	2.665^a^ (0.849)
Age _dummy (>55 years = 1, 0 = Otherwise)	−0.982^b^ (0.433)
Savings_dummy (No savings and below tk 5k = 1, 0 = Otherwise)	−1.577^a^ (0.473)
Total rice land (in decimal)	−0.007^a^ (0.002)
**Distributions of random coefficients**	
Standard deviation (Yield of rice)	0.254^a^ (0.042)
Standard deviation (Seed price of rice)	0.111^a^ (0.020)
**Model statistics**	
Parameters	16
Observations (N)	800
Pseudo R-squared	0.492
AIC	925.2
Log likelihood function	−446.595
AIC/N	1.156

*Note: Standard errors are reported in parentheses.*

^a^ *Significance at 1%*

^b^ *Significance at 5%*

^c^ *Significance at 10%*

In Table 3, the positive and significant coefficient for the Constant term indicates a strong preference to continue with traditional rice varieties, as the Constant is coded with the Status Quo option. The RPL model permits random taste heterogeneity in the sample population by enabling the utility coefficients to vary randomly across respondents [28]. In the RPL analysis, only two attributes (yield and seed price) were significant at randomised form. The positive random utility coefficient suggests that farmers in the sample, on average, perceive positive benefits from an increasing yield of rice. In contrast, the negative random utility coefficient suggests that farmers perceive negative benefits from an increasing price of rice seed. These results are consistent with prior expectations.

Preferences for improved rice varieties are influenced by interaction terms, making them complex. Both seed types (seed type 2 and seed type 3) have negative coefficients (Table 3), with the larger negative coefficient (−4.911) of the HYV variety of rice indicating that there is a stronger aversion to HYV rice than salt-tolerant rice. However, there are positive coefficients for interaction terms, suggesting that the preferences are more complex. The primary negative coefficients of seed type, seed price, and labor supplied indicate that farmers do not prefer improved seed types or higher seed prices, or more labor employed by themselves. However, the positive coefficients of interactions of seed type with seed price and labor indicate that farmers expect to pay more for seed type 2 and seed type 3 when combined with key inputs.

In the random parameter model, several socio-economic characteristics such as age, savings, and total rice land of the respondents were incorporated to enhance the model's ability to explain the data. The negative and significant coefficient of age implies that younger farmers are more likely to choose the status quo option, perhaps because younger farmers have lower savings and smaller land areas. Further, farmers with larger savings place a higher value on newer rice varieties. The negative and significant value of total rice land area suggests that smaller farmers are more sensitive to the choice impacts of newer rice varieties.

In line with the observed heterogeneity in preferences, the results from the latent class analysis (Table 4) reveal three classes of farmers with distinct preferences for rice variety attributes. Table 4 shows a noticeable variation in choice probabilities between the basic MNL model and the three groups from the latent class model. This MNL model represents the coefficients without explanatory variables. The values of the Pseudo R-squared statistic suggest that the LCM is a better fit than a basic MNL specification. Each class from LCM represents the choice responses quite differently with different attributes. The majority of respondents belong to class 3 (46.5%), followed by class 1 (33.2%) and class 2 (20.3%). It is worth noting that the coefficients are consistently higher in the LCM class 3 for all the attributes as compared with the other two classes, with the negative coefficients for seed type 2 and total labor supplied. The difference in valuations is particularly prominent for the seed type 3 attribute, with class 3 showing a positive benefit from salt-tolerant rice varieties. Moreover, the positive and significant coefficients of yield and seed price in class 3 indicate that those farmers are likely to pay more for salt-tolerant rice seed when there is a greater yield valuation. The class 1 and class 2 models did not yield significant coefficients for seed type, suggesting that these groups did not perceive benefits from adoption. It is noticeable from the LCM that all classes of farmers have positive coefficients for higher yields. A higher output price of rice and lower labor requirements contribute positive benefits for two groups of farmers (class 1 and class 3)

**Table 4:** Parameter estimates from MNL and Latent class models.

Variables	Multinomial logit model	Latent Class Model (LCM)		
		**Class 1**	**Class 2**	**Class 3**
Yield of rice	0.295^a^(.020)	0.187^a^(.030)	0.565^a^(0.106)	1.908^a^(0.460)
Seed type 2(Better HYV rice)	−0.422^b^(0.185)	0.202(0.267)	0.494(0.722)	−5.879^a^(2.073)
Seed type 3(Better salt-tolerant rice)	0.593^a^(0.190)	0.427(0.484)	−0.763(0.657)	6.216^a^(2.169)
Total labor supplied	−0.021^a^(0.008)	−0.026^b^(0.012)	0.099^a^ (0.029)	−0.231^c^ (0.121)
Seed price of rice	0.040^a^(0.013)	0.015(0.026)	−0.097^b^(0.044)	0.617^a^(0.180)
Rice output price	0.092^a^(0.020)	0.127^a^(0.040)	0.076(0.064)	0.400^b^(0.160)
ASC (Constant)	1.811^a^(0.277)	−0.370(0.606)	4.404^a^(1.184)	16.316^a^(4.326)
Latent class probabilities		0.332^a^(0.073)	0.203^a^(0.034)	0.465^a^(0.073)
**Model statistics**				
Log likelihood function	−524.58135	−425.281		
Pseudo R-squared	0.3857	0.516		
AIC	1063.2	896.6		
Observations (N)	800	800		
AIC/N	1.329	1.121		

*Note: Standard errors are reported in the parentheses;*

^a^ *Significance at 1%*

^b^ *Significance at 5%*

To examine whether the three preference segments identified through latent class analysis correspond to geographic salinity patterns, we analyzed the distribution of classes across the four sampled districts using post-hoc chi-square testing (Table 5). The chi-square test reveals no significant geographic clustering of preference classes (χ² = 2.893, df = 6, p = 0.822). The three latent classes are distributed relatively evenly across all four districts, with Class 1 comprising 30–36% of farmers in each district, Class 2 comprising 16–26%, and Class 3 comprising 38–52%. This pattern holds consistently across low-salinity (Patuakhali: 2–4 dS/m), moderate-salinity (Bagerhat: 4–8 dS/m), and high-salinity districts (Khulna and Satkhira: 6–12 dS/m), indicating that the preference heterogeneity captured by our Latent Class Model reflects individual-level differences in risk attitudes, resource constraints, and farming contexts rather than direct correlation with district-level salinity exposure patterns.

## 4. Discussions

The random parameters logit (RPL) model's results analysis yields several important conclusions about the factors influencing rice seed selection. It is true that better quality seeds often come at a higher price [99], and for this better quality seed, farmers are willing to pay more and hire more labor because of the potential benefits that they offer. High-quality seeds can result in higher crop yields and greater overall profitability for farmers. The finding of willingness to pay more for salt-tolerant rice is consistent with Arora et al. [28], who found similar results for drought and flood risks. High-quality seeds significantly increase yield and profitability in Bangladesh. Research demonstrates that quality seed management, including proper cleaning and treatment, can increase rice yields by 10–15% compared to farmer-saved seeds across different seasons and regions [100]. Field studies across 420 farmers in Northwest Bangladesh over two consecutive seasons demonstrated that improved varieties including Hybrid rice and BRRI dhan29 produced significantly higher yields (6.0–7.5 t/ha) compared to traditional varieties, with BRRI dhan29 showing the most stable yield and profitability performance [101]. These substantial productivity differentials explain farmers’ willingness to pay premium prices for certified seed when combined with favorable output prices. Farmers show a greater willingness to pay for maize and rice varieties that yield more under normal circumstances [27,102]. Several economic, environmental, and technological factors affect farmers’ willingness to pay for premium seeds. The perceived advantages, such as improved productivity, risk reduction, and fulfilling market requirements, contribute to the overall value proposition for farmers. Moreover, the interactions of total labor supplied with seed type 2 and seed type 3 are positive, which means that farmers are expected to employ more labor for seed type 2 and seed type 3 rice varieties. This suggests that certain improved varieties—particularly HYV and salt-tolerant varieties grown during the intensive Boro season—require more labor due to increased management demands. Irrigated Boro rice requires higher amounts of fertilizers, more frequent irrigation management, and careful monitoring for water stress, nutrient deficiencies, and optimal harvest timing to achieve their yield potential [101]. Salt-tolerant varieties in particular require careful water management during critical growth stages, additional weeding due to longer crop duration, and more intensive fertilizer application to compensate for nutrient uptake challenges under saline conditions [37]. The positive interaction coefficients between seed type and labor supply in our model (Table 3) suggest farmers recognize these management requirements and are willing to provide additional labor inputs when they perceive compensating yield benefits. In some way, the positive coefficients of the interaction term suggest that there is a diminishing effect on the relationship, putting a small quantity of curvature.

The findings from the latent class model (LCM) reveal significant differences in preferences among the three classes of respondents, particularly regarding the valuation of seed attributes. Class 3, which makes up 46.5% of the sample, consistently has higher coefficients for all attributes, suggesting a strong preference for improvements that boost resilience and productivity, particularly salt-tolerant rice varieties. This substantial group (46.5%) willing to invest in salt-tolerant varieties was identified through our Latent Class Model (LCM) analysis, which automatically segments respondents into distinct preference groups based on their choice patterns across the discrete choice experiment. The LCM algorithm endogenously determines the optimal number of classes and assigns farmers to groups based on their revealed preferences for different rice variety attributes, without any a priori classification by the researchers. This data-driven segmentation approach identifies latent (unobserved) heterogeneity in farmer preferences, with the 46.5% group representing farmers who consistently demonstrated a strong willingness to pay for salt-tolerant characteristics relative to other variety attributes in their choice responses. Class 1 (33.2%) represents farmers who do not show significant preferences for salt-tolerant varieties, prioritizing traditional attributes like low seed costs and labor requirements. Class 2 (20.3%) demonstrates limited willingness to invest in salt-tolerant varieties, focusing primarily on yield and profitability rather than salinity resistance.

These findings are consistent with previous research demonstrating significant heterogeneity in farmer preferences for agricultural technologies. Maligalig et al. [103] employed latent class analysis to examine Filipino rice farmers’ preferences for varietal trait improvements and identified four distinct farmer segments with varying priorities, including a stress tolerance-focused group (50%) and other groups prioritizing different attributes such as grain quality and pest resistance. Kostandini et al. [104] found heterogeneity in drought-tolerant variety preferences across East and Central Africa, with regional differences explaining variation. While our study identifies similar preference heterogeneity through latent class analysis, we did not test whether geographic location moderates preferences—a limitation noted above and important direction for future research. This pattern of preference heterogeneity, where a significant proportion of farmers do not prioritize stress-tolerant characteristics, aligns with broader literature on agricultural technology adoption, showing that farmer segments emerge naturally based on their distinct valuation of crop variety attributes [27].

The positive valuation of seed type 3 suggests a recognition of the long-term benefits associated with improved stress resistance, which may be crucial for farmers in regions facing salinity issues. Conversely, classes 1 and 2 may not fully comprehend or value the benefits of adopting such varieties, as indicated by the lack of significant coefficients for seed type in these groups. This suggests a possible awareness or perceived utility gap. This lack of significance could also suggest differing risk preferences or economic constraints that limit their willingness to invest in new seed types. Notably, while all classes displayed a positive inclination towards higher yields, only class 3 demonstrated a willingness to pay more for seed types with premium yields, particularly when accompanied by higher rice output prices and reduced labor inputs. This underscores the complexity of farmer decision-making, which is influenced not merely by market variables but also by class-specific characteristics and perceptions.Since adopting salt-tolerant varieties is economically viable, focused educational initiatives may help close the knowledge gap among the less receptive classes and eventually promote more sustainable agricultural practices.

Our study examines farmer preferences for salt-tolerant rice varieties among coastal farmers in salinity-affected regions (2–8 dS/m) without incorporating salinity level heterogeneity into the choice models. While our Latent Class Model identifies three distinct farmer segments with heterogeneous preferences—ranging from strong positive preferences (46.5%) to no significant preferences (33.2%) to limited engagement (20.3%)—we cannot determine whether this preference heterogeneity directly correlates with varying salinity severity levels farmers experience.

Specifically, we cannot assess whether farmers facing higher salinity value salt tolerance more intensely than those facing lower salinity, or whether the three identified preference classes systematically differ in their salinity exposure. Incorporating salinity level heterogeneity would have required: (1) information on salinity severity for individual farmers, (2) potentially redesigning the choice experiment to present varieties with specific tolerance thresholds corresponding to different salinity levels, and (3) modeling interactions between salinity exposure and preference parameters.

However, our findings remain directly applicable to their intended purpose: informing extension programs, breeding priorities, and adoption strategies for salt-tolerant varieties in coastal Bangladesh. Identifying three distinct farmer segments with differentiated preferences provides an actionable framework for targeted interventions. Understanding that nearly half of coastal farmers demonstrate strong willingness to invest in salt-tolerant varieties—while others require different support—represents crucial evidence for scaling climate-resilient technologies in salinity-affected regions.

Additionally, while we sampled across four districts with documented varying salinity levels to ensure representativeness across the coastal gradient, we did not incorporate district identifiers or salinity zone indicators as moderating variables in our models. This analytical decision reflects our focus on identifying preference segments among coastal farmers broadly rather than testing whether geographic location systematically influences preferences. In our panel data structure, each farmer's district assignment and all socio-demographic characteristics (age, savings, land size) remain constant across their four choice occasions. When combined with panel estimation, this creates perfect collinearity between individual fixed effects and time-invariant covariates, preventing estimation of coefficients for district or salinity zone variables in the Random Parameters Logit model.

To address whether the three latent classes correspond to geographic salinity patterns, we conducted post-hoc chi-square analysis testing whether class membership probabilities differ across districts. The results reveal no significant geographic clustering (χ² = 2.893, df = 6, p = 0.822), with the three preference segments distributed relatively evenly across low-salinity (Patuakhali), moderate-salinity (Bagerhat), and high-salinity districts (Khulna and Satkhira) (Table 5). This indicates that preference heterogeneity identified through our Latent Class Model operates largely independently of district-level salinity exposure, suggesting that factors such as risk attitudes, resource constraints, social networks, and farming experience—rather than salinity severity per se—drive the observed preference differentiation. Future research could examine whether farm-level salinity measurements (rather than district-level classifications) moderate preference intensity, or test whether other spatial factors such as proximity to extension services or market access influence preference formation.

## 5. Conclusion and policy recommendations

In relation to climate-induced salinity intrusion across coastal Bangladesh, this study reveals critical insights into farmer decision-making processes that are fundamental to designing effective climate adaptation strategies. Our analysis of farmer preferences for salt-tolerant rice varieties illuminates the balance between tradition and innovation that characterizes agricultural transformation in one of the world's most climate-vulnerable regions.

Our findings reveal farmers’ strong attachment to traditional rice varieties that transcends simple economic calculations. Despite scientifically superior alternatives, farmers continue to grow traditional rice varieties rooted in generations of accumulated knowledge. This preference may reflect rational responses to uncertainty where agricultural failure can mean destitution versus survival. Traditional varieties represent invaluable social capital that must be leveraged rather than dismissed in development interventions.

The Random Parameter Logit analysis reveals nuanced preference structures, where farmers exhibit a clear willingness to embrace innovation when specific conditions are met. The positive yield coefficient demonstrates that productivity gains serve as the primary catalyst for adoption—aligning with development objectives of enhancing agricultural productivity to reduce rural poverty. However, negative coefficients for seed price and labor requirements reveal how financial constraints create barriers even when technologies offer clear benefits. Crucially, interaction effects reveal farmers’ sophisticated cost-benefit calculations: willingness to pay premium prices for superior seeds demonstrates recognition of long-term value, while readiness to invest additional labor indicates understanding of input-outcome relationships. These findings reveal economically rational decision-making under uncertainty.

The latent class analysis provides actionable insights by revealing distinct farmer segments with differentiated preferences. The substantial group (46.5%) willing to invest in salt-tolerant varieties when yield benefits and output prices are favorable represents a critical entry point for scaling climate-resilient technologies. Widespread concern about additional labor requirements highlights a systemic constraint demanding policy attention.

Our findings provide an empirical foundation for designing climate adaptation strategies that simultaneously address productivity enhancement, poverty alleviation, and food security. The evidence offers policymakers analytical tools for moving beyond one-size-fits-all approaches toward nuanced interventions that leverage farmer heterogeneity to accelerate the adoption of salt-tolerant rice varieties. Based on our latent class findings, we propose differentiated adoption strategies. For high-willingness farmers (Class 3, 46.5%), who demonstrate strong preferences for salt-tolerant varieties but remain sensitive to seed costs, establish subsidized seed distribution centers and demonstration plots to accelerate adoption and create visible success stories. These early adopters can serve as supporters whose experiences build confidence among their peers. For uncertain farmers (Classes 1 and 2, 53.5%), who show limited or no significant preferences for salt-tolerant varieties, providing subsidized seed would be inefficient as seed access is not their primary barrier. Instead, these groups benefit more from free trial programs (small-scale, low-risk experimentation), farmer-to-farmer knowledge networks using Class 3 adopters as trusted information sources, and comprehensive technical training to address the root barriers of awareness and risk perception. This differentiated strategy maximizes impact: subsidies accelerate adoption among receptive farmers creating visible proof of concept, while knowledge-building interventions address fundamental barriers for uncertain farmers—ultimately expanding adoption more cost-effectively than uniform subsidies alone.

The strong positive response to output price improvements demands attention to market infrastructure and price stabilization. Developing robust value chains ensuring fair prices for improved varieties can create powerful adoption incentives while contributing to rural income growth. Consistent concern about labor requirements indicates urgent need for complementary mechanization strategies that reduce labor intensity while maintaining productivity advantages of improved varieties.

These recommendations face barriers including limited institutional capacity, resource constraints, market system weaknesses, and political challenges. Solutions require partnerships with government agencies for technical expertise, phased implementation prioritizing high-potential districts, co-financing with development partners, coordinated value chain development, and framing salt-tolerant rice within national food security policies. Extension programs should emphasize loss prevention rather than merely yield enhancement, as behavioral economics research demonstrates farmers are twice as sensitive to losses as gains. Specific risk mitigation support should include: (1) weather-indexed crop insurance providing automatic payouts when salinity or drought thresholds are exceeded, eliminating lengthy claim processes; (2) input credit for salt-tolerant seed with harvest-linked flexible repayment terms, protecting farmers from debt burden if performance is poor; (3) government or cooperative buy-back guarantees ensuring minimum output prices for salt-tolerant varieties, eliminating market risk for first-time adopters; (4) partial seed subsidies (e.g., 50% cost-sharing) reducing farmers’ financial exposure during experimentation; and (5) community demonstration plots where farmers can observe full-season performance before committing their own land, enabling vicarious learning without personal risk. These mechanisms directly address the loss aversion barriers identified in our behavioral economics framework while using successful local adopters as champions to build peer trust. Weather-indexed insurance implementation would require zone-specific threshold calibration using SRDI salinity and BMD meteorological data, adjusted for local conditions (e.g., 8 dS/m triggers in severe zones, 6 dS/m in moderate zones). Implementation could leverage existing SRDI monitoring networks, BMD stations, and satellite-based monitoring. Basis risk mitigation would require localized calibration at union/upazila level, multi-index combinations, and participatory design. Pilot testing would be essential. These operational considerations are beyond our current study scope but critical for implementation.

Future research should deepen understanding of socio-economic factors driving preference heterogeneity, particularly risk attitudes, social networks, and institutional contexts. Studies should examine how different framing approaches affect adoption decisions and explore behavioral economics mechanisms in agricultural technology adoption. Our methodological approach provides a replicable framework for understanding farmer preferences in climate-vulnerable regions worldwide, contributing to evidence-based policy design for climate-resilient agriculture.

## Supporting information

S1 File Choice modelling dataset. Contains the dataset used in the choice modelling analysis, including all variables and observations.
(XLSX)

S2 File Global research questionnaire. Includes the full questionnaire used for data collection in this study.
(DOCX)

S3 File Analytical results. Provides detailed outputs and supplementary analyses supporting the main results.
(PDF)

## Abbreviations

DCEs: Discrete Choice Experiments
MNL: Multinomial Logit Model
LCM: Latent Class Model
RPL: Random Parameter Logit model
