# Supplementary material for: Climate adaptation through farmer choice: Understanding preferences for rice varieties in coastal Bangladesh
Source: PLoS One. 2026 Apr 29;21(4):e0347541. doi: 10.1371/journal.pone.0347541 (PMC13128132; doi:10.1371/journal.pone.0347541)
Supplement: S2 File — Includes the full questionnaire used for data collection in this study. (DOCX) [file pone.0347541.s002.docx]

Inclusivity in global research

PLOS’ policy on inclusivity in global research aims to improve transparency in the reporting of research performed outside of researchers’ own country or community and ensures that PLOS publications reporting global research adhere to high standards for research ethics and authorship. Authors of relevant research articles may be asked to complete the questionnaire below, which outlines ethical, cultural, and scientific considerations specific to inclusivity in global research. This questionnaire may be requested when researchers have travelled to a different country to conduct research, if research uses samples collected in another country, research with Indigenous populations or their lands, or if research is on cultural artefacts. Researchers travelling to another country solely to use laboratory equipment will not normally be required to complete the questionnaire. However, the questionnaire can be requested at the journal’s discretion for any submission – if you have been requested to complete this questionnaire by the PLOS journal you submitted to, please do so.

Please complete the questionnaire below and include this as a Supporting Information file with your manuscript. Note that if your paper is accepted for publication, this checklist will be published with your article in the supporting information files. Please ensure that you reference the checklist in the main body of your manuscript. We suggest adding a subsection ‘Inclusivity in global research’ to your Methods section and adding the following sentence: “Additional information regarding the ethical, cultural, and scientific considerations specific to inclusivity in global research is included in the Supporting Information (SX Checklist)”

The questions have been designed to be applicable to a wide range of study types, and there are subsections for both human subjects research and non-human subjects research. If any of the questions are not relevant to your research please mark them as “N/A” as appropriate.

**Ethical considerations, permits and authorship**

*This section is applicable to all research types.*

Provide details as to who granted permissions and/or consent for the study to take place in the Methods section of your manuscript. This should include the names of **all** ethics boards, governmental organizations, community leaders or other bodies that provided approval for the study. If individuals provided approval refer to these people by their role or title but do not list their name(s).

Reported on page number: 20

If there were any deviations from the study protocol after approval was obtained please provide details of these changes in the Methods section of your manuscript.
Did this study involve local collaborators that are residents of the country where the research was conducted or members of the community studied? If you do not have any authors from said communities, please provide an explanation for this below.
Everyone listed as an author should meet PLOS’ criteria for authorship and all individuals who meet these criteria should be included in the author byline, rather than the acknowledgements. For further information please see the journal’s Authorship Policy.

Reported on page number: N/A

No, while the first author is a resident of Bangladesh (the country where research was conducted), no members of the specific rice farming communities studied are listed as co-authors. The study involved rice farmers as research participants rather than research collaborators.

The farmers were not included as co-authors for the following reasons:

• The research design employed a structured interview methodology where farmers served as respondents sharing their knowledge and experiences rather than as research partners in study design, analysis, or manuscript preparation

• The farmers expressed comfort participating as interview subjects rather than taking on formal research collaboration roles

• The study required specialized academic analysis and statistical interpretation that aligned with the research team's technical expertise

• However, meaningful engagement with the farming community was maintained through prior consultation with the local agricultural extension officer, conducting all interactions in Bengali, and plans to share findings back to the community through accessible summaries

• The first author's status as a Bangladeshi resident ensured cultural familiarity and appropriate engagement with local contexts, even though they are not specifically from the farming communities studied.

**Human subjects research (e.g. health research, medical research, cross-cultural psychology)**

Did you obtain written informed consent from a representative of the local community or region before the research took place? How did you establish who speaks for the community? Details of written informed consent obtained from study participants should be reported separately in the Methods section of your manuscript.

Written informed consent from a community representative was not obtained. However, verbal permission was secured from the local agricultural extension officer prior to engaging with rice farmers in the community.

The agricultural extension officer was identified as the appropriate community representative because:

• Extension officers serve as official government liaisons between agricultural departments and farming communities

• They have established relationships and credibility within local farming networks

• They possess authority to facilitate research access and understand community interests

• The officer confirmed that farmers would be willing to participate and that the research aligned with community interests

This approach was deemed appropriate given the rural context and established protocols for agricultural research in Bangladesh. Individual participant consent details are provided separately in the Methods section.

How did members of the local community provide input on the aims of the research investigation, its methodology, and its anticipated outcome(s)?

Community input was obtained through several pilot surveys conducted with rice farmers via telephone due to COVID-19 travel restrictions that prevented in-person engagement during the initial study design phase.

Community members provided input in the following ways:

• Research aims: During pilot surveys, farmers confirmed the relevance of investigating climate adaptation strategies through rice variety choices and validated that these issues were genuine concerns within their farming communities

• Methodology: The pilot surveys allowed farmers to provide feedback on interview questions, helping refine the questionnaire to ensure questions were culturally appropriate, clearly understood, and captured relevant farming practices and challenges

• Anticipated outcomes: Farmers expressed that research findings on “Climate adaptation through farmer choice: understanding preferences for rice varieties in coastal Bangladesh” would be valuable for improving their farming practices and informed the research team about what types of results would be most beneficial to their communities

While the COVID-19 pandemic limited initial face-to-face community consultation, the pilot survey process ensured that farmer perspectives informed the study design before full data collection commenced.

When engaging with the local community, how did you ensure that the informed consent documents and other materials could be understood by local stakeholders?

Several measures were taken to ensure that informed consent processes and interview materials could be understood by local rice farmers:

• Language: All consent processes and interviews were conducted in Bengali, the local language, eliminating language barriers

• Local data collectors: Data collectors were Bangladeshi nationals familiar with local dialects, cultural contexts, and farming terminology, ensuring effective communication with rural communities

• Verbal consent process: Given the rural context and varying literacy levels among farmers, verbal informed consent was used rather than written documents, allowing for interactive explanation and immediate clarification of any questions

• Comprehension verification: Data collectors confirmed participants' understanding by asking them to acknowledge key points about the study purpose, voluntary participation, and confidentiality before proceeding

• Cultural appropriateness: Interview questions and consent explanations were adapted to use farming terminology and concepts familiar to rice farmers, making the research purpose and process more accessible to the target community.

Will the findings of the research be made available in an understandable format to stakeholders in the community where the study was conducted (e.g. via a presentation, summary report, copies of publications, etc.)? Please provide details of how this will be achieved.

The research findings have been presented at an international conference, but direct dissemination to the participating farming communities has not yet been completed due to travel to Bangladesh. However, we recognize the importance of community benefit sharing and plan to present the key results to farmer groups through extension office meetings.

**Non-human subjects research using specimens/ animals collected as part of the study, or those housed in archival collections. Examples include archaeology, paleontology, botany and zoology.**

Did the permission you obtained from a local authority to perform the study include an agreement on access to outputs and benefit sharing? This may include procedures to enable fair distribution of the benefits and resources arising from the research performed. Please include any details of Prior Informed Consent and Benefit Sharing Agreements obtained. These may be required by field-specific regulations, for example the Convention on Biological Diversity (CBD) and the associated Nagoya Protocol.

N/A

If the material used in your study was imported, please A) provide the year it was imported and B) indicate whether permits were obtained to import/export the materials used, C) provide details of any permits obtained. If this information is not available, please indicate this.

N/A

If you used archival specimens, please state how the material used in your study was acquired by the institute it is held in and provide details of any permits obtained for the original excavations/ sample collection. If this information is not available, please indicate this.

N/A

How was the potential cultural significance of the materials collected in your study to local communities considered in your research design? Were Indigenous peoples and/or local researchers and institutions involved with archaeological excavations / collection of specimens? If so, please provide a description of their involvement.

N/A

If your manuscript includes photographs of human remains please indicate whether authors obtained permission from descendants or affiliated cultural communities to do so.

N/A
