# Supplementary material for: Climate adaptation through farmer choice: Understanding preferences for rice varieties in coastal Bangladesh
Source: PLoS One. 2026 Apr 29;21(4):e0347541. doi: 10.1371/journal.pone.0347541 (PMC13128132; doi:10.1371/journal.pone.0347541)
Supplement: S3 File — Provides detailed outputs and supplementary analyses supporting the main results. (PDF) [file pone.0347541.s003.pdf]

The results from all econometric models

```
Start values obtained using MNL model
Dependent variable                Choice
Log likelihood function       -494.44699
Estimation based on N =    800, K =  14
Inf.Cr.AIC  =   1014.8 AIC/N =    1.271
---------------------------------------
            Log likelihood R-sqrd R2Adj
Constants only   -853.9701  .4165 .4043
Note: R-sqrd = 1 - logL/Logl(constants)
Warning:  Model does not contain a full
set of ASCs. R-sqrd is problematic. Use
model setup with ;RHS=one to get LogL0.
Root Likelihood:Geom. Mean of P^  .5387
---------------------------------------
Response data are given as ind. choices
Number of obs.=   800, skipped    0 obs
--------+-------------------------------------------------------------
----
        |                  Standard            Prob.      95% Confidence
  CHOICE|  Coefficient       Error       z     |z|>Z*         Interval
--------+-------------------------------------------------------------
----
  BYEILD|     .28217***      .02046    13.79  .0000      .24407     .32427
 BSPRICE|    -.04114         .02909    -1.41  .1573     -.09815     .01587
 BSEED2D|   -3.16644***      .68548    -4.66  .0000    -4.62876   -1.92211
 BSEED3D|    -.74836         .57261    -1.31  .2148    -1.83266     .41193
 BLABOUR|    -.04060***      .01397    -2.91  .0037     -.06799    -.01322
  BPRICE|     .11583***      .02192     5.29  .0000      .07288     .15879
 BSD2SPR|     .19711***      .04807     4.10  .0000      .10989     .29833
 BSD3SPR|     .07395*        .04032     1.83  .0743     -.00707     .15097
BLABSED2|     .04704*        .02545     1.85  .0646     -.00284     .09693
BLABSED3|     .04113*        .02117     1.94  .0521     -.00037     .08263
     ASC|    1.98665***      .51802     3.81  .0000     1.43507    3.83023
    BAGE|    -.54613**       .24540    -2.22  .0124     -.34979    -.04248
BNO_LOWS|    -.90123***      .23093    -3.91  .0000    -1.43471    -.51774
BTOTALRI|    -.00453***      .00134    -3.37  .0007     -.00717    -.00190
--------+-------------------------------------------------------------
----
***, **, * ==>  Significance at 1%, 5%, 10% level.
Model was estimated on Nov 17, 2022 at 03:07:05 PM
----------------------------------------------------------------------
----

Estimation results: matrix LASTOUTP or LASTPRTL in project.
Iterative procedure has converged
Normal exit:  27 iterations. Status=0, F=    .4460359D+03

----------------------------------------------------------------------
----
Random Parameters Multinom. Logit Model
Dependent variable                CHOICE
Log likelihood function       -446.59587
Restricted log likelihood     -878.88983
Chi squared [ 16](P= .000)     865.70793
Significance level                .00000
McFadden Pseudo R-squared       .4925008
Estimation based on N =    800, K =  16
Inf.Cr.AIC  =    925.2 AIC/N =    1.156
```

```
---------------------------------------
            Log likelihood R-sqrd R2Adj
No coefficients  -878.8898  .4925 .4871
Constants only   -853.9701  .4777 .4721
At start values  -494.8830  .0987 .0890
Note: R-sqrd = 1 - logL/Logl(constants)
Root Likelihood:Geom. Mean of P^  .5726
Warning:  Model does not contain a full
set of ASCs. R-sqrd is problematic. Use
model setup with ;RHS=one to get LogL0.
---------------------------------------
Response data are given as ind. choices
Replications for simulated probs. =1000
Used Halton sequences in simulations.
RPL model with panel has     200 groups
Fixed number of obsrvs./group=        4
Number of obs.=   800, skipped    0 obs
--------+------------------------------------------------------------------
----
        |                 Standard            Prob.      95% Confidence
  CHOICE|  Coefficient       Error       z    |z|>Z*         Interval
--------+------------------------------------------------------------------
----
        |Random parameters in utility functions............................
  BYEILD|     .50387***       .05194     9.70  .0000       .39607     .59966
 BSPRICE|    -.09955**        .04391    -2.27  .0234     -.18561    -.01348
        |Nonrandom parameters in utility functions.........................
 BSEED2D|   -4.91156***     1.00922    -4.87  .0000    -6.95259  -2.99653
 BSEED3D|   -1.98786**        .83456    -2.31  .0211    -3.52985    -.28587
 BLABOUR|    -.05888***       .02061    -2.86  .0043     -.09928    -.01849
  BPRICE|     .18864***       .03353     5.62  .0000      .11913     .25015
 BSD2SPR|     .28064***       .06679     4.25  .0000      .15274     .41454
 BSD3SPR|     .15626**        .05927     2.64  .0104      .03505     .26348
BLABSED2|     .06566*         .03528     1.86  .0627     -.00348     .13481
BLABSED3|     .07756**        .03088     2.56  .0104      .01799     .13513
     ASC|    2.66578***       .84970     3.14  .0005     1.56735    5.65021
    BAGE|    -.98209**        .43396    -2.27  .0397     -.57427    -.01390
BNO_LOWS|   -1.57755***       .47336    -3.36  .0005    -2.58916    -.71795
BTOTALRI|    -.00679***       .00226    -3.01  .0026     -.01122    -.00237
        |Distns. of RPs. Std.Devs or limits of triangular.................
NsBYEILD|     .25391***       .04193     6.05  .0000      .16474     .32909
NsBSPRIC|     .11108***       .02029     5.47  .0000      .07228     .14788
--------+------------------------------------------------------------------
----
***, **, * ==>  Significance at 1%, 5%, 10% level.
Model was estimated on Nov 17, 2022 at 03:23:42 PM
---------------------------------------------------------------------------
----

Estimation results: matrix LASTOUTP or LASTPRTL in project.
|-> SAVE;file="C:\Windows\System32\Choice modelling2.lpj"$
```

4

5

```
--------------------------------------------------------------------------
----

Discrete choice (multinomial logit) model
Dependent variable               Choice
Log likelihood function      -524.58135
Estimation based on N =    800, K =   7
Inf.Cr.AIC  =   1063.2 AIC/N =    1.329
---------------------------------------
            Log likelihood R-sqrd R2Adj
Constants only   -853.9701  .3857 .3768
Note: R-sqrd = 1 - logL/Logl(constants)
Warning:  Model does not contain a full
set of ASCs. R-sqrd is problematic. Use
model setup with ;RHS=one to get LogL0.
Root Likelihood:Geom. Mean of P^  .5191
---------------------------------------
Response data are given as ind. choices
Number of obs.=   800, skipped    0 obs
--------+-----------------------------------------------------------------
----
        |                  Standard            Prob.      95% Confidence
  CHOICE|  Coefficient       Error       z    |z|>Z*         Interval
--------+-----------------------------------------------------------------
----
BYEILD|1|     .29494***      .01961    15.04  .0000      .25650    .33337
BSEED2|1|    -.42194**       .18547    -2.27  .0229     -.78546   -.05842
BSEED3|1|     .59294***      .19003     3.12  .0018      .22048    .96540
BLABOU|1|    -.02058***      .00768    -2.68  .0074     -.03564   -.00552
BSPRIC|1|     .03956***      .01269     3.12  .0018      .01469    .06444
BPRICE|1|     .09214***      .01993     4.62  .0000      .05308    .13120
   ASC|1|    1.81071***      .27734     6.53  .0000     1.26713   2.35428
--------+-----------------------------------------------------------------
----
***, **, * ==>  Significance at 1%, 5%, 10% level.
Model was estimated on Nov 21, 2022 at 01:26:17 PM
--------------------------------------------------------------------------
----

Estimation results: matrix LASTOUTP or LASTPRTL in project.
Iterative procedure has converged
Normal exit:  76 iterations. Status=0, F=    .4252809D+03

--------------------------------------------------------------------------
----
Latent Class Logit Model
Dependent variable               CHOICE
Log likelihood function      -425.28093
Restricted log likelihood    -878.88983
Chi squared [ 23](P= .000)    907.21780
Significance level               .00000
McFadden Pseudo R-squared      .5161158
Estimation based on N =    800, K =  23
Inf.Cr.AIC  =    896.6 AIC/N =    1.121
---------------------------------------
            Log likelihood R-sqrd R2Adj
No coefficients  -878.8898  .5161 .5091
Constants only   -853.9701  .5020 .4947
```

6

```
At start values  -524.5966  .1893 .1775
Note: R-sqrd = 1 - logL/Logl(constants)
Root Likelihood:Geom. Mean of P^  .5877
Warning:  Model does not contain a full
set of ASCs. R-sqrd is problematic. Use
model setup with ;RHS=one to get LogL0.
---------------------------------------
Response data are given as ind. choices
Number of latent classes =            3
Average Class Probabilities
     .332  .203  .465
LCM model with panel has     200 groups
Fixed number of obsrvs./group=        4
Number of obs.=   800, skipped    0 obs
--------+---------------------------------------------------------------
----
        |                  Standard            Prob.      95% Confidence
  CHOICE|  Coefficient       Error       z    |z|>Z*         Interval
--------+---------------------------------------------------------------
----
        |Random utility parameters in latent class -->>  1...............
BYEILD|1|     .18683***      .02997     6.23  .0000      .12809    .24557
BSEED2|1|     .20156         .26677      .76  .4499     -.32130    .72443
BSEED3|1|     .42697         .48371      .88  .3774     -.52108   1.37503
BLABOU|1|    -.02639**       .01163    -2.27  .0233     -.04919   -.00359
BSPRIC|1|     .01538         .02616      .59  .5566     -.03589    .06664
BPRICE|1|     .12697***      .03959     3.21  .0013      .04938    .20457
   ASC|1|    -.36979         .60577     -.61  .5416    -1.55707    .81750
        |Random utility parameters in latent class -->>  2...............
BYEILD|2|     .56478***      .10642     5.31  .0000      .35620    .77336
BSEED2|2|     .49404         .72156      .68  .4935     -.92020   1.90827
BSEED3|2|    -.76328         .65681    -1.16  .2452    -2.05060    .52404
BLABOU|2|     .09877***      .02941     3.36  .0008      .04114    .15640
BSPRIC|2|    -.09703**       .04407    -2.20  .0277     -.18341   -.01066
BPRICE|2|     .07609         .06405     1.19  .2348     -.04943    .20162
   ASC|2|    4.40446***     1.18410     3.72  .0002     2.08366   6.72525
        |Random utility parameters in latent class -->>  3...............
BYEILD|3|    1.90752***      .45984     4.15  .0000     1.00624   2.80880
BSEED2|3|   -5.87838***     2.07343    -2.84  .0046    -9.94223  -1.81453
BSEED3|3|    6.21639***     2.16911     2.87  .0042     1.96502  10.46776
BLABOU|3|    -.23051*        .12136    -1.90  .0575     -.46837    .00736
BSPRIC|3|     .61698***      .18006     3.43  .0006      .26406    .96990
BPRICE|3|     .40024**       .16045     2.49  .0126      .08578    .71471
   ASC|3|    16.3159***     4.32642     3.77  .0002      7.8363   24.7955
        |Estimated latent class probabilities............................
 PrbCls1|     .33200***      .07316     4.54  .0000      .18862    .47539
 PrbCls2|     .20305***      .03412     5.95  .0000      .13617    .26992
 PrbCls3|     .46495***      .07276     6.39  .0000      .32234    .60756
--------+---------------------------------------------------------------
----
***, **, * ==>  Significance at 1%, 5%, 10% level.
Model was estimated on Nov 21, 2022 at 01:26:19 PM
------------------------------------------------------------------------
----

Estimation results: matrix LASTOUTP or LASTPRTL in project.
|-> SAVE;file="C:\Windows\System32\Choice modelling2.lpj"$
```

7

8
